# Interpretable machine learning model for identification and risk factor of premature rupture of membranes (PROM) and its association with nutritional inflammatory index: a retrospective study

**DOI:** 10.3389/fmed.2025.1557919

**Published:** 2025-06-18

**Authors:** Meng Zheng, Xiaowei Zhang, Haihong Wang, Ping Yuan, Qiulan Yu

**Affiliations:** Department of Obstetrics and Gynecology, Binhai County People’s Hospital, Yancheng, Jiangsu, China

**Keywords:** PROM, machine learning, nomogram, nutritional inflammation index, predictive models

## Abstract

**Background:**

Premature rupture of membranes (PROM) poses significant risks to both maternal and neonatal health. This study aims to construct a risk factor prediction model related to PROM by using machine learning technology and explore the association with nutritional inflammatory index.

**Methods:**

A retrospective analysis was conducted on patients with PROM. Based on the variables screened out by ridge regression and Boruta algorithm, univariate and multivariate logistic regression analyses were further adopted. According to the sample data, it is divided into the training set and the internal validation set in a ratio of 7:3. The research group adopted four machine learning algorithms: Extreme Gradient Boost (XGBoost), Support Vector Machine (SVM), Logistic Regression (LR), and Random Forest (RF). The selected variables were incorporated into model construction, with the area under the receiver operating characteristic (ROC) curve (AUC) serving as a criterion for model selection. Model performance was assessed using AUC values, sensitivity, specificity, recall, F1 score, and accuracy. The variables were selected based on the contribution degree of the variables in Shapley additive Interpretation (SHAP) to construct the nomogram.

**Results:**

A retrospective analysis was conducted involving 800 parturients at Binhai County People’s Hospital from January 2023 to October 2024, comprising 400 with PROM and 400 with normal delivery. The RF model demonstrated superior performance with an AUC of 0.757, sensitivity of 67.4%, and specificity of 65.1%. Key predictive factors identified included body mass index (BMI), prognostic nutritional index (PNI), platelet, albumin, and aggregate index of systemic inflammation (AISI). The ROC of the model also showed good efficacy, with an AUC of 0.777.

**Conclusion:**

This study highlights the potential of machine learning in enhancing the understanding and prediction of PROM, and emphasizes the significance of inflammatory and nutritional indicators, paving the way for future research in maternal-fetal medicine.

## 1 Introduction

Premature rupture of membranes (PROM) is a well-known risk factor for preterm birth, with an incidence rate as high as 10%, accounting for 30%–40% of all preterm births (1). PROM is a significant obstetric complication characterized by the rupture of fetal membranes before the onset of labor. This condition can lead to increased risks for both maternal and neonatal morbidity and mortality, including infections and adverse fetal outcomes. Pregnant women may suffer from placental abruption, intrauterine infection, puerperal infection and postpartum hemorrhage, while newborns may suffer from fetal distress, NRDS, intracranial hemorrhage and septicemia, and the morbidity and mortality of newborns in the perinatal period are significantly increased (2). The pathophysiological mechanisms underlying PROM are complex and multifactorial, involving factors such as infection, inflammation, and mechanical stress on the membranes. Reproductive tract infections have been shown to affect the structural integrity and function of the fetal membrane, thereby promoting the occurrence of premature PROM (3, 4). Understanding the risk factors associated with PROM is crucial for developing preventive strategies and optimizing management to improve outcomes for both mothers and infants.

Systemic inflammation is characterized by changes in neutrophilia, lymphocytopenia, and thrombocytosis (5). However, the individual sensitivity of these parameters is quite poor, so various indices containing combinations of parameters have been developed. Neutrophil and lymphocyte ratio (NLR), lymphocyte and monocyte ratio (LMR), platelet and lymphocyte ratio (PLR), systemic immune inflammation index (SII) and Prognostic Nutritional Index (PNI) indicators have been studied as new inflammatory markers in many diseases (6–9). One study demonstrated the prognostic significance of NLR, PLR, MLR, and SII in predicting latency in patients with PROM (10). In recent years, studies have found a significant association between the systemic inflammatory response index (SIRI) and pregnancy-related pathological conditions (such as gestational hypertension, PROM, etc.) suggesting that it may play an important role in the inflammatory response and related complications during pregnancy (10, 11). The NLR in advanced lung cancer inflammation index (ALI) reflects the systemic inflammatory state, while the occurrence of PROM may be related to intrauterine infection or inflammatory response. Theoretically, the systemic inflammatory state may affect pregnancy outcomes, but currently there is a lack of direct evidence (12). Aggregate index of systemic inflammation (AISI) has the potential to predict admission to the neonatal intensive care unit and chorioamnionitis in pregnant women with PROM (13).

In recent years, the emergence of machine learning techniques in the field of obstetrics has paved the way for enhanced prediction models aimed at identifying risk factors associated with adverse pregnancy outcomes (14). Understanding the predictive factors for PROM can inform clinical decision-making and improve outcomes for affected patients. A notable gap in the research remains regarding the integration of machine learning methodologies in the identification of risk factors specifically associated with PROM. Traditional statistical methods, while valuable, often fall short in handling the complexity of high-dimensional data. In contrast, machine learning algorithms have demonstrated superior performance in various medical applications by effectively managing such complexities and enhancing predictive accuracy (15, 16).

This research endeavors to harness the capabilities of machine learning to advance our understanding of the risk factors associated with PROM, with the ultimate goal of improving patient outcomes through informed clinical decision-making. By bridging the gap between computational techniques and clinical application, we hope to contribute to the evolving landscape of obstetric care and precision medicine.

## 2 Materials and methods

### 2.1 Study population

From January 2023 to October 2024, a total of 800 cases of women giving birth in the obstetrics department were collected in Binhai County People’s Hospital, of which 400 were women giving birth normally and 400 were women with PROM. The study was approved and supervised by the Ethics Committee of Binhai County People’s Hospital (Approval No. 2024-BYKYLL-016) and was conducted in accordance with the standards of the Declaration of Helsinki (revised in 2013).

The inclusion criteria are as follows:

(1) Parturients aged ≥ 18 years old, with singleton pregnancies, and who delivered in the hospital during the gestation period from ≥ 28 weeks to < 42 weeks;
(2) Parturients who meet the diagnostic criteria for PROM and those with normal delivery.

Exclusion criteria:

(1) Pregnancy comorbidities and complications, such as severe maternal diseases (preeclampsia, placental abruption, placenta previa with hemorrhage), and other diseases that may interfere with the analysis of inflammatory indicators;
(2) Within 2 weeks before delivery, there is a clear history of bacterial/viral infection (such as positive Group B Streptococcus, positive TORCH [Toxoplasma; Other; Rubella virus; Cytomegalovirus; Herpessimplex virus)-Immunoglobulin M(IgM)] or treatment with antibiotics/immunosuppressants;
(3) Combined with autoimmune diseases (such as systemic lupus erythematosus, rheumatoid arthritis, etc.) or immune deficiency status like acquired Immune Deficiency Syndrome(AIDS);
(4) Missing data and information, incomplete laboratory indicators or basic personal information;
(5) Pregnant women with malignant tumors.

### 2.2 Clinical data collection

Our study included the following variables: (1) Basic information: age, smoking and alcohol consumption; (2) Laboratory indicators: white blood cell (WBC), neutrophil count, lymphocyte count, monocyte count and platelet count; (3) Underlying diseases: hypertension and diabetes; (4) Pregnancy status: whether it is multiple pregnancy and whether it is multiparous. The laboratory indicators for this study were collected by trained professionals and the biochemical components were analyzed using the same automated system, performed by the laboratory physician and scored by the specialist. Previous diseases and pregnancy status were mainly determined by the patient’s medical history and B-ultrasound examination.

Requirements for the collection of laboratory indicators: PROM group: All blood samples were collected from the last routine prenatal examination before the occurrence of PROM, and met the following conditions: collected before the occurrence of PROM, and no antibiotics (such as cephalosporins), glucocorticoids (such as dexamethasone), or uterine contraction induction therapy were received; There were no signs of infection from the prenatal examination to the occurrence of PROM. Exclude those who received vaginal procedures or immunomodulatory drugs after prenatal examinations. Normal delivery group: Blood samples were selected from pregnant women with matched gestational ages (± 1 week) and no PROM. They were collected from routine prenatal examinations at the same gestational age (such as examinations at 36 weeks of pregnancy), and met the following conditions: no uterine contractions were initiated and no analgesic drugs were used; No PROM occurred within at least two weeks after the prenatal examination (confirmed by follow-up).

### 2.3 Calculation of nutritional inflammation index

Neutrophil-to-lymphocyte ratio, LMR, PLR, SII, PNI, SIRI, ALI and AISI indicators are calculated as follows: NLR = neutrophil count/lymphocyte count; LMR = Lymphocyte count/monocyte count; PLR = Platelet count/lymphocyte count; SII = (neutrophil count × platelet count)/lymphocyte count; PNI = albumin + 5 × lymphocyte count; SIRI = Neutrophil count × monocyte count/lymphocyte count; ALI = body mass index (BMI) × serum albumin level/NLR; AISI = platelet count × neutrophil count × monocyte count/lymphocyte count. Due to the right-biased distribution of AISI, AISI underwent a logarithmic transformation (Ln) in the regression analysis.

### 2.4 Statistical analysis

Baseline characteristics of all included patients were stratified according to whether PROM had occurred. Variables that did not conform to the normal distribution were represented as the quartile range, and the Mann-Whitney U test was used to compare differences between groups. Categorical variables are expressed as percentages and compared using chi-square tests. Firstly, features are selected based on ridge regression analysis and the Boruta algorithm. This method selects important features through compression coefficients and reduces dimensions, screens out features with greater contributions and eliminates redundant features. Subsequently, based on the variables screened out by ridge regression and the Boruta algorithm, univariate and multivariate logistic regression analyses were further adopted to select the variables with statistical differences. Meanwhile, restricted cube plots (RCS) and threshold effect analysis were adopted to screen the conceptuality between variables and TPROM. Subsequently, the sample data were divided into the training set and the internal validation set in a ratio of 7:3. The team used four machine learning algorithms: extreme Gradient Boosting (XGBoost), support vector machines (SVM), logistic regression (LR), and Random Forest (RF). The final selected variables were incorporated into the model construction. In selecting the best model, we use the maximum area under the curve (AUC) of the receiver operating characteristic curve (ROC) in the verification set as the evaluation basis. The performance of the predictive model was evaluated by the AUC value, sensitivity, specificity, recall rate, F1 score and accuracy under the ROC of the training set and validation set. The higher the value of AUC, the better the ability of the model to distinguish. Sensitivity and specificity reflect the ability of the model to correctly identify positive and negative samples, respectively. Recall rates and F1 scores were combined to account for the sensitivity and specificity of the model. In addition, decision curve analysis (DCA) and calibration curves were plotted to demonstrate true clinical utility. Using the Shapley additive explanations (SHAP) method, a bar graph was plotted to show the contribution of each feature to the predicted results. SHAP evaluation of selected cases shows the impact of specific features on a particular sample and helps us understand the decision-making process of the model. Finally, based on the screened variables, we constructed the nomogram to enable readers to have a more intuitive understanding of the factors causing premature rupture of membranes. All statistical analyses were performed using R software (version 4.3.0) and STATA 17.0 (64-bit), with bilateral *p*-values < 0.05 considered statistically significant.

## 3 Results

### 3.1 Demographic and clinical characteristics

A total of 800 parturients were included in this study, of which 400 (50%) were normal parturients and 400 (50%) were PROM. Table 1 shows the baseline information. Compared with normal parturients, the levels of neutrophils, NLR, SII and AISI of parturients with PROM were significantly higher, with statistical significance (all *p* < 0.05). The indexes of lymphocytes, platelets, albumin, LMR, PLR, PNI and ALI in parturients with PROM were lower than those in normal parturients, and the differences were statistically significant (all *p* < 0.05). PROM was more likely to occur in multiple pregnancies, multiparous, people with hypertension, diabetes, smoking habits and drinking habits (all *p* < 0.05). However, there was no statistical significance for age.

**TABLE 1:** Baseline demographic and clinical characteristics of parturients were included.

Characteristic	Total no. (%)	Non-PROM	PROM	*P*-value
		No. (%)	No. (%)	
Total	800	400 (50)	400 (50)	–
Age (years)	29.00 (26.00, 33.00)	30.00 (25.00, 34.00)	29.00 (26.00, 32.00)	0.206
BMI kg/m^2^	23.2 (21.3, 24.5)	22.5 (20.8, 23.9)	23.9 (22.1, 26.8)	< 0.001
WBC	6.9 (5.9, 8.1)	6.9 (5.9, 8.2)	6.9 (5.9, 8.0)	0.385
Neutrophil	6.75 (5.59, 8.54)	6.63 (5.21, 8.35)	6.91 (5.80, 8.78)	< 0.001
Lymphocyte	1.49 (1.19, 1.78)	1.51 (1.25, 1.85)	1.45 (1.13, 1.69)	< 0.001
Monocyte	0.660.55, 0.84)	0.68 (0.53, 0.85)	0.65 (0.55, 0.83)	0.524
Platelet	201 (162, 241)	210. (167, 251)	193 (159, 228)	< 0.001
Albumin	34.70 (32.900, 36.30)	35.000 (33.40, 36.70)	34.30 (32.20, 35.80)	< 0.001
NLR	4.57 (3.59, 6.53)	4.30 (3.21, 5.71)	4.890 (3.840, 7.59)	< 0.001
LMR	2.25 (1.69, 2.81)	2.26 (1.81, 2.94)	2.23 (1.44, 2.72)	< 0.001
PLR	132.35 (108.11, 177.00)	135.71 (106.16, 179.34)	130.49 (110.70, 176.03)	0.797
SII	922.03 (662.12, 1358.78)	908.04 (635.74, 1270.16)	948.78 (710.70, 1485.45)	0.002
PNI	42.000 (39.60, 44.25)	42.65 (40.50, 45.30)	41.300 (38.55, 43.25)	< 0.001
ALI	17.2 (11.9,23.0)	18.0 (13.4, 24.1)	16.2 (10.4, 22.0)	< 0.001
SIRI	6.6 (4.4, 10.6)	6.7 (4.4, 10.9)	6.5 (4.4, 10.4)	0.996
AISI	6.4 (6.0, 7.0)	6.4 (5.9, 6.8)	6.5 (6.1, 7.1)	< 0.001
Parity, *n* (%)				0.023
No	636 (79.50)	331 (82.75)	305 (76.25)	–
Yes	164 (20.50)	69 (17.25%)	95 (23.75)	–
Multiple_PregnTancy, *n* (%)				0.004
No	776 (97%)	395 (98.75)	381 (95.25)	–
Yes	24 (3%)	5 (1.25%)	19 (4.75)	–
Hypertension, *n* (%)				< 0.001
No	717 (89.62)	375 (93.75)	342 (85.50)	–
Yes	83 (10.37)	25 (6.25)	58 (14.50)	–
Diabetes, *n* (%)				0.002
No	741 (92.62)	382 (95.50)	359 (89.75)	0.002
Yes	59 (7.37)	18 (4.50)	41 (10.25)	–
Drinking, *n* (%)				0.032
No	676 (84.50)	349 (87.25)	327 (81.75)	–
Yes	124 (15.50)	51 (12.75)	73 (18.25)	–
Smoking, *n* (%)				0.016
No	740 (92.50)	379 (94.75)	361 (90.25)	–
Yes	60 (7.50)	21 (5.25)	39 (9.75)	–

### 3.2 Feature selection

**FIGURE 1:**
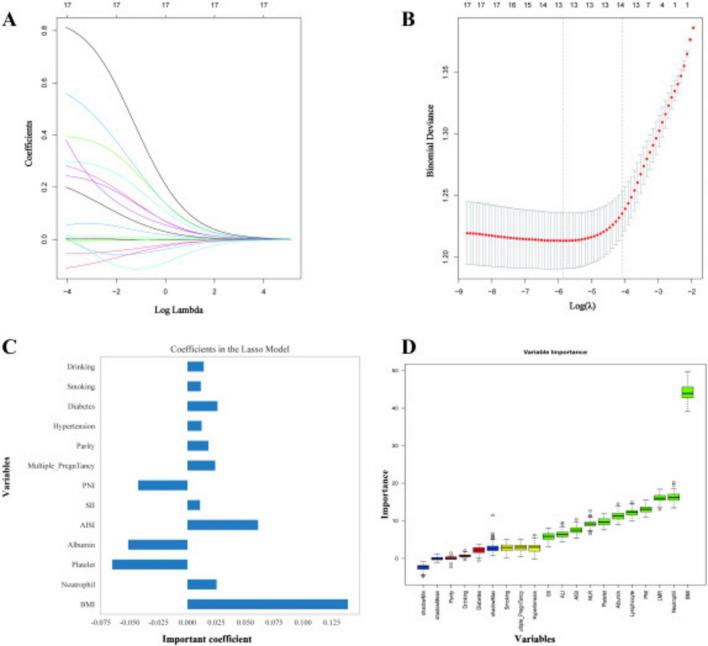
Ridge regression analysis. **(A–C)** A 10–fold cross–validation iteration was used to determine the penalty coefficient, and the variables that significantly contributed to premature rupture of membranes at term were screened through standardized coefficients; **(D)** Fourteen key variables were confirmed through the importance score.

Based on the indicators with statistically significant differences in the previous baseline characteristic analysis, this study optimized variable selection through a dual-feature screening strategy. Ridge regression analysis: To solve the problem of multicollinearity, a 10-fold cross-validation iteration was adopted to determine the penalty coefficient, and the variables that significantly contributed to PROM at term were screened through standardized coefficients (Figures 1A–C). Ultimately, 13 variables were included: BMI, neutrophils, platelets, albumin, SII, PNI, AISI, multiple pregnancies, multiparas, diabetes, hypertension, alcohol consumption and smoking. Boruta algorithm screening: 500 iterations were conducted, and 14 key variables were confirmed through importance scores (Figure 1D): BMI, neutrophils, LMR, PNI, lymphocytes, albumin, platelets, NLR, AISI, ALI, SII, hypertension, multiple pregnancies, and smoking. Retain the 10 variables jointly identified by the two algorithms (such as BMI, neutrophils, platelets, albumin, SII, PNI, AISI, hypertension, multiple pregnancies, and smoking); Add four immune-related indicators unique to the Boruta algorithm (LMR, lymphocytes, NLR, ALI).

### 3.3 Univariate and multivariate analysis of nutritional inflammation index and TROM

Based on the 14 variables screened above, in order to further clarify their relationship with PROM, the research group conducted univariate and multivariate logistic regression analyses (Table 2). Univariate logistic regression analysis indicated that albumin, lymphocytes, platelets, ALI, LMR, and PNI were all protective factors. The remaining variables were all risk factors, with multiple pregnancies being the most significant. Subsequently, multivariate logistic regression analysis was further used for variable screening. There were statistically significant differences in BMI (OR = 1.29, 95% CI 1.20–1.39), albumin (OR = 0.83, 95% CI 0.78–0.89), platelets (OR = 0.99, 95% CI 0.98–0.99), AISI (OR = 2.73, 95% CI 1.13–6.58) and PNI (OR = 0.83, 95% CI 0.78–0.89).

**TABLE 2:** Univariate analysis and multivariate analysis of premature rupture of membranes (PROM).

Variables	Univariate analysis		Multivariate analysis	
	OR (95% CI)	*P*	OR (95% CI)	*P*
**Multiple pregnancy**				
No	1.00 (Reference)	–	1.00 (Reference)	–
Yes	3.94 (1.46∼10.66)	0.007	2.72 (0.93∼7.95)	0.067
**Hypertension**				
No	1.00 (Reference)	–	1.00 (Reference)	–
Yes	2.54 (1.56∼4.16)	< 0.001	1.68 (0.93∼3.02)	0.083
**Smoking**				
No	1.00 (Reference)	–	1.00 (Reference)	–
Yes	1.95 (1.13∼3.38)	0.017	1.52 (0.81∼2.87)	0.196
BMI	1.30 (1.23∼1.38)	< 0.001	1.29 (1.20∼1.39)	**< 0.001**
Albumin	0.84 (0.80∼0.89)	< 0.001	0.83 (0.78∼0.89)	**< 0.001**
Neutrophil	1.12 (1.06∼1.18)	< 0.001	0.99 (0.86∼1.13)	0.852
Lymphocyte	0.44 (0.32∼0.59)	< 0.001	0.79 (0.36∼1.78)	0.574
Platelet	0.99 (0.98∼0.99)	< 0.001	0.99 (0.98∼0.99)	**< 0.001**
ALI	0.97 (0.96∼0.99)	< 0.001	1.00 (0.95∼1.06)	0.914
AISI	1.58 (1.30∼1.93)	< 0.001	2.73 (1.13∼6.58)	**0.025**
NLR	1.15 (1.09∼1.20)	< 0.001	0.92 (0.82∼1.03)	0.169
LMR	0.70 (0.61∼0.81)	< 0.001	1.27 (0.88∼1.84)	0.195
SII	1.01 (1.01∼1.01)	< 0.001	1.00 (1.00∼1.00)	0.151
PNI	0.85 (0.82∼0.89)	< 0.001	0.83 (0.78∼0.89)	**< 0.001**

The bold values means *p* < 0.05.

### 3.4 Non-linear associations between platelets, albumin, AISI, PNI, and PROM

**FIGURE 2:**
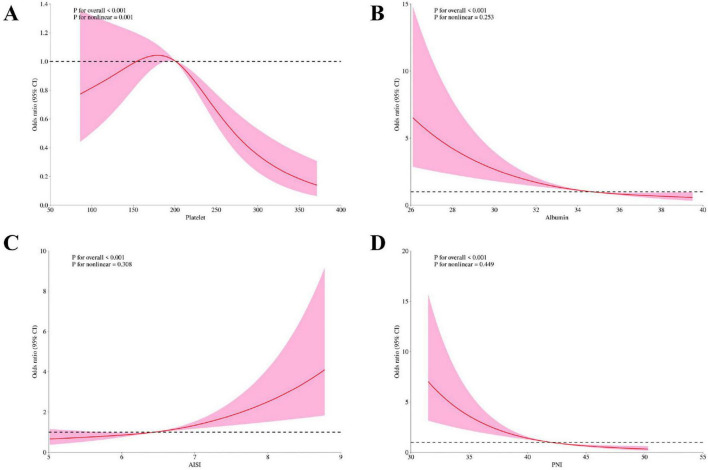
Restricted cube plots (RCS) between platelets, albumin, aggregate index of systemic inflammation (AISI) and prognostic nutritional index (PNI) and premature rupture of membranes (PROM). **(A)** platelets; **(B)** albumin; **(C)** AISI; **(D)** PNI.

To deeply explore the non-linear dose-response relationship between platelets, albumin, AISI and PNI and PROM, this study used RCS and piecewise regression models for analysis (Figure 2 and Table 3). We observed that platelets had an “L” -shaped relationship with the occurrence of PROM (Figure 2A); Albumin and PNI levels were negatively correlated with the outcome (Figures 2B, C), and positively correlated with AISI (Figure 2D), but not completely linearly correlated. Through threshold effect analysis (Table 3), BMI and AISI were positively correlated with the outcome, and both had threshold effects. The inflection points were 24.81 and 7.694, respectively. Among them, when BMI was higher than 24.81, OR (95% CI): 5.76 (2.10–15.80). When AISI was lower than 7.694, OR (95% CI): 1.36 (1.08–1.71), and there were no statistically significant differences in the rest. Platelet, albumin and PNI also have threshold effects and are negatively correlated. The inflection points are 289.375, 30.003, and 48.152, respectively. When platelet is higher than 289.375, OR (95% CI): 0.97 (0.94–0.99), when Albumin was higher than 30.003, OR (95% CI): 0.88 (0.82–0.94) and when PN was lower than 48.152, OR (95% CI): 0.87 (0.83–0.91).

**TABLE 3:** Analyze the threshold effect of aggregate index of systemic inflammation (AISI) and other indicators on premature rupture of membranes through two segmented regression models.

Variables	Adjusted OR (95% CI)	*P*-value
**BMI**		
Fitting by the standard linear model	1.30 (1.23–1.38)	<0.001
Inflection point	24.81	−
BMI < 24.81	1.08 (1.00–1.17)	0.061
BMI > 24.81	3.76 (2.10–5.80)	<0.001
Log likelihood ratio	−	<0.001
**Platelet**		
Fitting by the standard linear model	0.98 (0.95–0.99)	<0.001
Inflection point	289.37	−
Platelet < 289.37	1.00 (0.99–1.00)	0.121
Platelet > 289.37	0.97 (0.94–0.98)	0.023
Log likelihood ratio	−	0.001
**Albumin**		
Fitting by the standard linear model	0.84 (0.80–0.89)	<0.001
Inflection point	30.00	−
Albumin < 30.00	0.48 (0.23–1.01)	0.054
Albumin > 30.00	0.88 (0.82–0.94)	< 0.001
Log likelihood ratio	−	0.017
**AISI**		
Fitting by the standard linear model	1.58 (1.30–1.93)	<0.001
Inflection point	7.69	−
AISI < 7.69	1.36 (1.08–1.71)	0.010
AISI > 7.69	2.63 (0.65–3.98)	0.093
Log likelihood ratio	−	0.019
**PNI**		
Fitting by the standard linear model	0.85 (0.82–0.89)	<0.001
Inflection point	48.15	−
PNI < 48.15	0.87 (0.83–0.91)	<0.001
PNI > 48.15	0.29 (0.06–1.30)	0.105
Log likelihood ratio	−	0.048

### 3.5 Multi-model comparison

**FIGURE 3:**
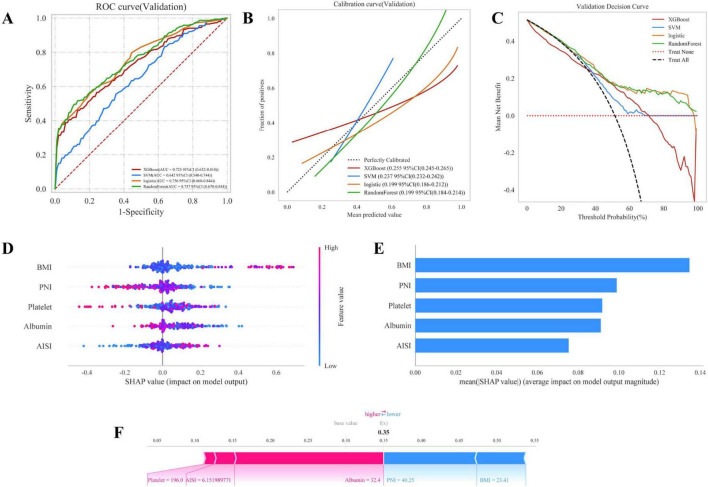
Four machine learning models are used to identify the risk factors of PROM. **(A)** The discriminatory performance of the four models in terms of the receiver operating characteristic (ROC) curve; **(B)** Calibration curves of the four models; **(C)** decision curve analysi (DCA); **(D,E)** Comprehensive group maps; **(F)** Detailed case.

Based on the screening of the above variables, in order to obtain the best model, we constructed four machine learning models to identify the risk factors of PROM in parturients (Figure 3). Figure 3A shows the discriminatory performance of these four models in terms of the ROC curve. The four models with PROM all exhibited considerable effects in predictive performance, among which the RF model performed the best. The AUC of the four models are as follows: Logistic: 0.756, RF: 0.757, SVM: 0.642 and XGBoost: 0.725. Among them, all three models have good predictive capabilities, but they are ranked in descending order of performance as follows: The RF model is the best, followed by the Logistic, XGBoost and SVM models. Table 4 shows the detailed performance indicators of the four models. The RF model demonstrated superior overall performance (training set sensitivity: 0.721, specificity: 0.683; validation set sensitivity: 0.674, specificity: 0.651). It is notable that both the accuracy rate and the F1 value of the RF model are the highest, with the accuracy rate being (training set: 0.698; validation set: 0.661) and F1 value (training set: 0.694; verification set: 0.670). Meanwhile, the calibration curves of the four models are presented in Figure 3B, demonstrating the good consistency between the predicted probabilities of the RF model and the observed results. Figure 3C shows the DCA, and the result remains consistent with the previous one. It is found that the DCA of the RF model is the best.

**TABLE 4:** Performances of the machine learning models for predicting premature rupture of membranes (PROM).

Model	Train/test	Sensitivity	Specificity	F1 score	Accuracy	Auc
Logistic	Train	0.589 (0.521–0.639)	0.849 (0.788–0.893)	0.651 (0.604–0.693)	0.693 (0.653–0.746)	0.793 (0.728–0.882)
	Test	0.535 (0.500–0.569)	0.809 (0.748–0.870)	0.624 (0.587–0.660)	0.668 (0.631–0.704)	0.757 (0.670–0.844)
RF	Train	0.721 (0.684–0.774)	0.683 (0.631–0.762)	0.694 (0.656–0.724)	0.698 (0.665–0.776)	0.798 (0.707–0.867)
	Test	0.674 (0.624–0.717)	0.651 (0.590–0.712)	0.67 (0.646–0.694)	0.661 (0.635–0.686)	0.756 (0.668–0.844)
XGBoost	Train	0.587 (0.429–0.645)	0.735 (0.670–0.780)	0.593 (0.568–0.618)	0.668 (0.646–0.694)	0.754 (0.664–0.836)
	Test	0.505 (0.465–0.535)	0.715 (0.640–0.750)	0.593 (0.568–0.618)	0.648 (0.636–0.660)	0.725 (0.632–0.818)
SVM	Train	0.532 (0.394–0.653)	0.656 (0.417–0.745)	0.506 (0.397–0.615)	0.572 (0.552–0.612)	0.683 (0.583–0.764)
	Test	0.498 (0.288–0.693)	0.626 (0.377–0.721)	0.506 (0.397–0.615)	0.555 (0.530–0.581)	0.642 (0.540–0.744)

### 3.6 Interpretability analysis

Figures 3D, E present a comprehensive population graph, illustrating the variables in the RF model. The horizontal axis represents the SHAP value, while the vertical axis shows the features that are ranked according to their cumulative SHAP values. Each data point corresponds to a specific instance, and its position on the X-axis represents the SHAP value of that specific instance and feature. The results showed that the contribution proportions of each variable, in descending order, were: BMI, PNI, platelets, albumin and AISI. Figure 3F provides a detailed case study, demonstrating the prediction process of the model for a specific patient. In this visualization, the red indicator represents the negative contribution to the prediction, while the blue indicator represents the positive impact. The f(x) value represents the actual SHAP value of each factor.

### 3.7 Construction of the nomogram

**FIGURE 4:**
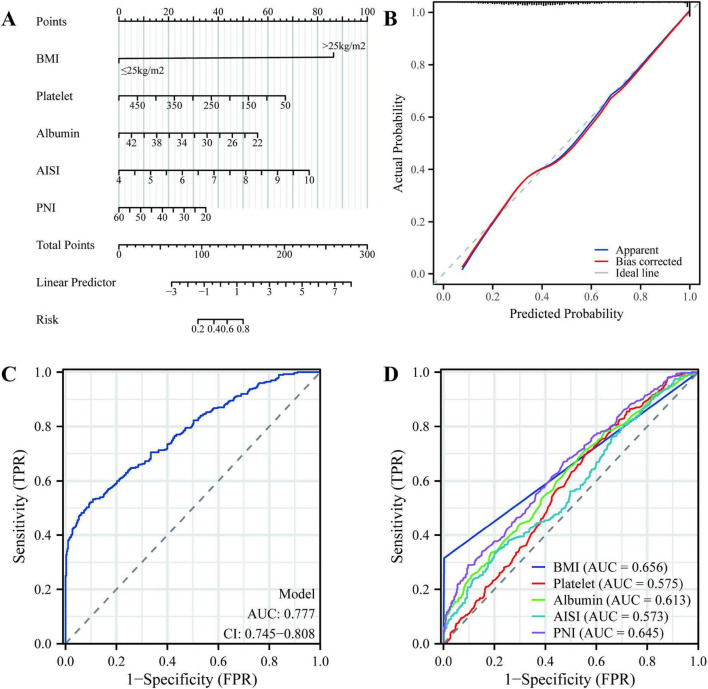
The construction of the nomogram. **(A)** Nomogram model; **(B)** Calibration curve; **(C)** Receiver operating characteristic (ROC) of the model; **(D)** ROC of five individual variables.

In order to enhance the clinical applicability of the model and facilitate the rapid decision-making of clinicians, we constructed the nomogram model based on the above screening variables (Figure 4A). These five variables include: BMI, PNI, platelets, albumin and AISI. The ROC of the model also showed good efficacy, with an AUC of 0.777 (Figure 4C). Meanwhile, the calibration curve was plotted (Figure 4B), demonstrating good consistency. Meanwhile, the research group also plotted the ROC of five individual variables, and their AUC were respectively: BMI: 0.656, PNI:0.645, platelet: 0.575, albumin: 0.613 and AISI: 0.573 (Figure 4D). According to the above results, it shows that the model of the research group has a better predictive efficiency compared with other single factors.

## 4 Discussion

So far, the pathogenesis of PROM has been unclear, often as a result of multifactorial interactions. Once the membrane ruptures, the barrier protection immediately disappears. Pregnant women will irreversibly be subjected to amniotic infection, chorioamniotic inflammation, fetal distress and placental abruption until preterm delivery (17). Although the causes of PROM are varied, infection and inflammation are often highlighted as important factors. During pregnancy, the level of progesterone in the body of women increases, promoting the increase of vaginal secretions and vaginal mucosal congestion, thus providing necessary conditions for bacterial reproduction. According to statistics, the incidence of bacterial vaginitis in pregnant women is 10%–50% (18).

In this retrospective study, we developed a machine learning-based model to identify risk factors associated with PROM and constructed an effective predictive framework. One of the significant innovations of this research lies in the utilization of multiple machine learning algorithms, including XGBoost, SVM, LR, and RF, alongside Lasso regression for feature selection. This combination not only enhances the robustness of our model but also provides a comprehensive approach to understanding the underlying factors contributing to PROM. Previous studies have primarily focused on traditional statistical methods, often neglecting the advantages that machine learning can offer in terms of predictive power and interpretability. Our findings align with existing literature that emphasizes the importance of integrating advanced computational techniques to improve clinical decision-making in obstetrics. Furthermore, our analysis demonstrated that the use of SHAP provided valuable insights into feature contributions to the predictive model. This method effectively illustrated the impact of individual variables on the likelihood of PROM occurrence, enhancing the interpretability of the model’s predictions. By focusing on the five factors: BMI, PNI, platelets, albumin, and AISI, we promote a more intuitive understanding of the risk factors involved, which is critical for clinicians to make informed decisions about prenatal care.

A previous study showed that blood routine examination was a good predictor of premature rupture of membranes, but only leukocyte had statistical significance between the PROM group and the normal group (19). Systemic inflammation characterized by neutrophilism, lymphocytopenia, and thrombocytosis can be evaluated with a simple blood test. However, the reliability of a single parameter in identifying inflammation is low. Therefore, it is necessary to combine the indices of multiple inflammatory parameters. It is of great significance to incorporate inflammatory nutritional index into the prediction of risk factors for PROM. Our study found that neutrophils, NLR, SII, and AISI in PROM were significantly higher, while lymphocytes, platelets, albumin, LMR, PLR, PNI, and ALI were lower, and the differences were statistically significant. During pathogen invasion and infection, neutrophils are stimulated to secrete a series of pro-inflammatory cytokines, regulatory cytokines, and chemokines, thereby inducing an inflammatory cascade. Conversely, lymphocytes, which are a major component of the immune system, suppress the inflammatory response of the body by secreting anti-inflammatory factors, such as IL-10 (20). Monocytes are a crucial component of the innate immune response. After their recruitment, monocytes continuously secrete pro-inflammatory cytokines, enzymes, and growth factors (21). A study by Cappelletti et al. (22) determined that placental inflammation is a result of histological chorioamnionitis, which can be predicted by NLR. Kim et al. (23) found that NLR had a high predictive value for placental inflammation. PLR is more valuable in inflammatory and thrombotic diseases, but it may be affected by pregnant women with gestational diabetes, acute pancreatitis, preeclampsia, or PROM (24). Tanacan et al. (25) demonstrated that SII levels in PROM pregnant women were positively correlated with adverse neonatal outcomes. However, NLR, LMR, PLR and SII only evaluate the occurrence of the systemic inflammatory response induced by the activation of immune function and do not consider the immune and nutritional status of the individuals. PNI, which is an important parameter that reflects the nutritional and inflammatory state of the body, is calculated using serum albumin level and lymphocyte count. In recent years, studies have highlighted a close relationship between PNI and the prognosis of various tumors, myocardial infarction, and congenital heart diseases (9, 26). ALI reflects the systemic inflammatory state, while the occurrence of PROM may be related to intrauterine infection or inflammatory response. Theoretically, the systemic inflammatory state may affect pregnancy outcomes, but currently there is a lack of direct evidence (12). AISI is an emerging systemic inflammatory indicator, aiming to comprehensively assess the inflammatory status of the body. It provides a more comprehensive assessment of inflammation by integrating the levels of multiple inflammatory factors. This indicator is used clinically to predict various pathological conditions, especially in the health management of pregnant women and newborns. The physiological significance of AISI lies in that it can reflect the overall response of the body to inflammatory stimuli, thereby providing clinicians with important information about the severity and prognosis of the disease (27). The research results show that the AISI value is significantly higher in pregnant women who need to be admitted to the neonatal intensive care unit than in those who do not. Moreover, in patients with chorioamnionitis, the AISI value is also significantly higher than that in patients without chorioamnionitis (13).

The monitoring of BMI has important clinical significance during pregnancy. Studies have shown that BMI levels during pregnancy are closely related to a variety of adverse pregnancy outcomes. Both too low and too high BMI are associated with an increased risk of complications such as PROM, preterm birth, and gestational hypertension (28, 29). Obesity is regarded as a low-grade chronic inflammatory state. Adipose tissue in the body secretes various pro-inflammatory factors, such as tumor necrosis factor-α (TNF-α), interleukin-6 (IL-6), etc. These factors may affect pregnancy outcomes, including PROM (30). Hypertension and diabetes are significant risk factors for PROM in pregnant women. Abnormal cytotrophoblast invasion and endothelial dysfunction of spiral arterioles can lead to placental ischemia and placental endothelial injury (31, 32). Elevated blood glucose levels affect the expression of metalloproteinases in the fetal membrane, leading to dehydration of the membrane cells and fibrinogen, which ultimately weakens the tensile strength of the fetal membrane. At the same time, multiple pregnancies and multiparous may cause cervical muscle damage, increase the likelihood of cervical incompetence, and subsequently develop reproductive tract infections after pregnancy, and increase the risk of PROM (33). Smoking and drinking in pregnant women are risk factors for PROM. Nicotine in tobacco constricts blood vessels, increases carbon monoxide levels in the body, and causes PROM to occur when the mother and fetus are deprived of oxygen. Alcohol tends to constrict placental blood vessels, affecting fetal blood supply, resulting in fetal membrane dysplasia, resulting in PROM (34).

However, we acknowledge that our study has certain limitations that are worth discussing. Limitations of this study include its retrospective design, which may introduce selection bias and limit the generality of our findings. In addition, our study is limited to a single institution, which may affect the external validity of the predictive models developed. Reliance on historical data can also result in incomplete or inaccurate clinical records, potentially affecting the accuracy of identifying risk factors. Future studies should aim to validate these findings in larger, multicenter coves to improve the reliability of predictive models and explore the integration of real-time clinical data. Addressing these limitations is critical to advancing the role of machine learning in obstetric practice and improving outcomes for women at risk of PROM.

## 5 Conclusion

In conclusion, our research successfully identified the key risk factors related to PROM through machine learning methods and explored the relationship between the inflammatory nutrition index and PROM, providing a valuable predictive model for clinical applications. By evaluating multiple machine learning algorithms, we build a model with strong discriminative ability, as can be seen from the AUC value. The application of SHAP analysis further illuminates the contribution of individual characteristics and enhances our understanding of the underlying mechanisms. These findings highlight the potential of machine learning in obstetric practice, paving the way for more targeted risk assessment and management strategies in clinical Settings.

## Data Availability

The raw data supporting the conclusions of this article will be made available by the authors, without undue reservation.
